# Time to address the double inequality of differences in dietary intake between Scotland and England

**DOI:** 10.1017/S0007114518001435

**Published:** 2018-07-28

**Authors:** Karen L. Barton, Stephanie Chambers, Annie S. Anderson, Wendy L. Wrieden

**Affiliations:** 1 Division of Food and Drink, Abertay University, Dundee DD1 1HG, UK; 2 MRC/CSO Social and Public Health Sciences Unit, University of Glasgow, Glasgow G2 3QB, UK; 3 Centre for Public Health Nutrition Research, University of Dundee, Dundee DD1 9SY, UK; 4 Human Nutrition Research Centre and Institute of Health and Society, Newcastle University, Newcastle upon Tyne NE2 4HH, UK

**Keywords:** Food consumption, Nutrient intakes, Scotland, England, Inequalities

## Abstract

Geographical disparities in health outcomes have been evident across the UK for decades. Recent analysis on the dietary differences between Scotland and England that might go some way to explain these health differences is limited. This study aimed to assess whether, and to what degree, aspects of diet and nutrition differ between Scottish and English populations, specifically between those with similar household incomes. A period of 12 years of UK food purchase data (2001–2012) were pooled and used to estimate household-level consumption data for Scotland and England. Population mean food consumption and nutrient intakes were estimated, adjusting for known confounders (year, age of household reference person, age they left full-time education and income). Comparison was also made within equivalised income quintiles. Analysis showed that the foods and nutrients that should be increased in the diet (highlighted in the Scottish Dietary Goals) were lower in Scotland than in England (e.g. fruit and vegetables 267 g/d; 99 % CI 259, 274 *v.* 298 g/d; 99 % CI 296, 301), *P*<0·001). Similarly, foods and drinks linked with poor health outcomes were higher in Scotland. These regional inequalities in diet were even more pronounced in the lower-income groups (e.g. red and processed meat consumption in the lowest-income quintile was 65 g/d; 99 % CI 61, 69 in Scotland *v*. 58 g/day; 99 % CI 57, 60 in England, *P*<0·001, but similar in the highest-income quintile (58 g/d; 99 % CI 54, 61 *v*. 59 g/d; 99 % CI 58, 60, respectively). A poorer diet in Scotland compared with England, particularly among disadvantaged groups, may contribute to differences in excess mortality between countries.

Over 20 years ago, a Scottish Office report on the Scottish Diet^(^ ^1^ ^)^ concluded that dietary patterns in Scotland were contributing to high rates of chronic diseases such as heart disease, obesity, type 2 diabetes, high blood pressure, stroke and some cancers. The longstanding recognition of the need to tackle poor diet and obesity in Scotland led to the Scottish Office publishing the Scottish Diet Action Plan and setting the Scottish Dietary Targets in 1996^(^ ^2^ ^)^, revised in 2013 and 2016 as the Scottish Dietary Goals^(^ ^3^ ^,^ ^4^ ^)^.

Progress towards these targets has been slow, and there has been a failure to eliminate inequalities in dietary patterns^(^ ^5^ ^–^ ^7^ ^)^. Such inequalities have been acknowledged in key policy documents as contributing to the risk of chronic diseases, including obesity^(^ ^8^ ^,^ ^9^ ^)^. However, it is not only within Scotland that dietary inequalities are of concern: dietary inequalities between Scotland and England have been put forward as an explanation for excess mortality in Scotland compared with England^(^ ^10^ ^)^. These differences cannot be explained by deprivation alone^(^ ^11^ ^)^. Of particular concern is that those with low socio-economic status living in Scotland might be experiencing a double impact of dietary inequalities by consuming diets of poorer nutritional value than their fellow citizens in Scotland with higher socio-economic status^(^ ^7^ ^)^, as well as those with similar socio-economic status in England. One illustration of this is the higher percentage of those achieving 5-a-d in England in the lowest-income quintile but not in the highest-income quintile in the National Diet and Nutrition Survey (NDNS) year 1–4 results for the UK compared with Scotland^(^ ^12^ ^,^ ^13^ ^)^.

The reason for differing food choices between Scotland and England are complex but undoubtedly relate to culture, tradition and perceived acceptability of energy-dense foods and drinks now celebrated and marketed as part of the cultural heritage (e.g. deep fried Mars Bar, Irn Bru (sugar-containing soft drink) and processed meats including haggis and black pudding)^(^ ^1^ ^)^.

A review of the literature^(^ ^14^ ^)^ identified significant historical differences in the intake of foods and nutrients, which are likely to have health implications^(^ ^15^ ^)^, between Scotland and England or between Scotland and the Northern regions of England. In Scotland, there was lower fruit and vegetable consumption, lower fibre intake, lower intake of most water- and fat-soluble vitamins and a higher intake of Na. The information in these studies and reports was somewhat limited, however, by small sample sizes for Scotland^(^ ^16^ ^,^ ^17^ ^)^, lack of rigour in dietary assessment methods and lack of appropriate statistical analysis controlling for confounding factors^(^ ^14^ ^)^.

Recent work by Scarborough *et al*.^(^ ^10^ ^,^ ^18^ ^)^ modelled the change in population mortality from CHD, stroke and ten diet-related cancers that would be expected given a change in the average dietary quality within a population. Using this, they have shown that improvements in diet towards current dietary guidelines are associated with reduction in disease mortality, and in particular that if Scotland achieved an average diet equivalent in nutritional quality to the average diet in England then 40 % of the excess cardiovascular and cancer mortality would be removed. They compared food and nutrient data from just 3 years of the Family Food Survey annual reports and data sets (2007, 2008 and 2009) published by the UK Department for Environment, Food and Rural Affairs (Defra), and found that average diets in Scotland contained more fat, saturated fat and salt, and less fruit and vegetables. A lower consumption of vegetables and fibre (NSP) was also found in the comparison of diet between Scotland and the whole of the UK using recent NDNS data^(^ ^12^ ^)^.

The food and nutrient data used in the annual Family Food reports^(^ ^19^ ^)^, the Living Costs and Food Survey (LCFS), has a sample size of about 500 households in Scotland per year and has been used to monitor dietary trends, as well as socio-economic differences in diet, using both area-based measures of deprivation and equivalised income^(^ ^7^ ^,^ ^20^ ^)^. It is therefore a suitable source of data to explore differences that exist between Scotland and England in equally deprived areas or sectors of the population, something that was not reported by Scarborough *et al*.^(^ ^10^ ^)^.

The Revised Scottish Dietary Goals^(^ ^3^ ^)^ set out in nutritional terms ‘the diet that will improve and support the health of the Scottish population’. They are based on established national and international guidelines and are set at the Scottish population level, and include fruit and vegetables, oily fish, red and processed meat, energy, fats, sugar, salt and fibre. ‘They indicate the direction of travel, and assist policy development to reduce the burden of obesity and diet-related disease in Scotland’. It is therefore appropriate to focus any exploration of differences that exist between Scotland and England and equally deprived areas or sectors of the population in relation to the foods and nutrients specified by these goals.

This study sought to use food purchase data to examine nutritional differences for Scotland and England more robustly and extending the comparison made by Scarborough *et al*.^(^ ^10^ ^,^ ^18^ ^)^ by combining annual data from the UK food purchase survey from 2001 to 2012 and adjusting for waste (both edible and inedible) as in previous work by Barton and colleagues^(^ ^6^ ^,^ ^7^ ^,^ ^20^ ^)^. The aims of this study were to assess whether, and to what degree, aspects of diet and nutrition differ between Scottish and English populations and specifically between those with similar household incomes living in Scotland and England.

## Methods

A period of 12 years (2001–2012) of data from the annual UK food purchase survey, the Expenditure and Food Survey (EFS)/LCFS^(^ ^21^ ^)^, were used to explore comparisons of dietary intake for Scotland and England. The EFS/LCFS is a continuous household purchase (budget) survey, which collects household food purchase data over a 14-d period. Detailed information on *Survey Sampling for Family Food* ^(^ ^22^ ^)^ and *Quality and Methodology Information* ^(^ ^23^ ^)^ has been published by Defra and the Office for National Statistics, respectively. Sampling of the EFS/LCFS is designed in such a way to ensure that the results are representative of the population of the UK^(^ ^23^ ^)^. The survey data are weighted to reduce the effect of non-response bias and produce population totals and means. Response rate for the EFS/LCFS has reduced over the 12 years of the study from 62 % in 2001 to 50 % in 2010; however, rates appeared to stabilise and increased again to 54 and 52 % for 2011 and 2012, respectively.

Methods used in previous work to compare population mean consumption were used^(^ ^6^ ^)^. Estimates of food consumption and nutrient intakes were calculated from household food and eaten out purchases following secondary analysis to convert purchase data to mean per capita food consumption and nutrient intakes. Purchase data were adjusted for waste using estimates of edible waste published by Waste and Resource Action Programme Survey^(^ ^24^ ^)^. These have been mapped by Defra to each of the food codes used in the EFS/LCFS and were used to assign a waste factor to each food code. The waste figures were provided for single and multiple adult households and were linked to the appropriate type of household before analysis. Inedible waste (i.e. bone) was also taken into account when calculating the adjustment factor for each food code. The mean daily nutrient intake per person was calculated on the basis of the nutrient content of the foods/drinks, adjusted for waste, divided by the number of individuals in the household. The EFS/LCFS food composition database from Defra was used to calculate nutrient intake (the data for this being supplied by the Department of Health from the NDNS).

Univariate Analysis of Variance – Weighted Least Squares Regression (SPSS version 20; SPSS Inc.) was used to obtain means, 99 % CI and an indication of statistical significance (*P*<0·01) for differences by country (Scotland *v*. England)^(^ ^25^ ^)^. Results are presented as population per capita means (i.e. including consumers and non-consumers). Unadjusted and adjusted analyses were carried out. The multivariate model used in the analysis further adjusted by survey year, age of household reference person, age when the household reference person left full-time education* and equivalised income quintile* (*proxies for socio-economic position). As the survey collects data at the household level, it was not possible to adjust for individual ages or sex; however, the data are weighted to account for different household types. The EFS/LCFS sample was split into annual equivalised income quintiles at the UK level, and the 12 years of data were recombined to allow comparison between Scotland *v*. England, within each equivalised income quintile (adjusted for survey year, age of household reference person and age when the household reference person left full-time education).

### Ethical statement

This study was conducted according to the guidelines laid down in the Declaration of Helsinki. As the study was secondary analysis of anonymised data, ethical approval was not required.

## Results

Table 1 presents results for 2013 Scottish Dietary Goals foods and nutrients obtained from the analysis of EFS/LCFS data from 2001 to 2012 combined (excluding Na, which can only be accurately measured from urinary output). Results for further foods and nutrients are available in Supplementary Tables S1 and S2. For Scotland *v*. England, analysis showed that population-adjusted mean fruit and vegetable and oil-rich fish consumption was lower for Scotland, and red and processed meat intake was higher. No difference was found for energy intake or fat as a percentage of food energy. However, the percentage of energy from saturated fat and non-milk extrinsic sugars (NMES) (added sugars, sugar in fruit juice and half of the sugar naturally present in fruit that is canned, stewed, dried or used in preserves) was higher for Scotland than for England, and fibre (as NSP) intake was lower. The differences in these nutrients were reflected in the foods that contribute to them. Hence, consumption of whole milk, butter, processed meat (reported as other red meat and bacon and ham), processed potatoes, savoury snacks and sugar-containing soft drinks was higher in Scotland compared with that in England (online Supplementary Table S2). Intakes of vitamins A, D and E and folate were lower in Scotland but intake of Cawas higher (online Supplementary Table S1).

**Table 1:** Consumption of 2013 Scottish Dietary Goal foods and nutrients, 2001 to 2012 combined, Expenditure and Food Survey/Living Costs and Food Survey data (Mean values and 99 % confidence intervals)

	Scotland (6431 households)		England (59 958 households)		
Foods or nutrients	Mean†	99 % CI	Mean†	99 % CI	*P*
Energy (MJ/d)	8·4	8·3, 8·5	8·3	8·3, 8·4	0·070
Fruit and vegetables (g/d)‡ §	267	259, 274	298	296, 301	<0·001*
Fruit (g/d)‡	141	136, 145	150	149, 151	<0·001*
Vegetables (g/d)§	126	122, 130	148	147, 150	<0·001*
Oil-rich fish (g/week)	30·0	27·3, 32·6	35·4	34·6, 36·3	<0·001*
Total red and processed meat (g/d)||	62·0	60·4, 63·6	59·4	58·9, 60·0	<0·001*
% Food energy – fat	38·8	38·9, 39·1	38·9	38·8, 39·0	0·412
% Food energy – saturated fat	15·2	15·1, 15·3	14·9	14·9, 14·9	<0·001*
% Food energy – non-milk extrinsic sugars¶	15·2	15·0, 15·4	14·8	14·7, 14·9	<0·001*
Fibre (NSP) (g/d)**	12·3	12·1, 12·5	12·8	12·8, 12·9	<0·001*

* *P*<0·01 considered significant.

† Means adjusted by survey year, equivalised income quintiles, age of household reference person, age when the household reference person left full-time education.

‡ Fruit includes fruit and vegetable juice.

§ Vegetables include baked beans.

|| Meat portion only – see appendices 2 and 4 of Barton *et al.* ^(^ ^34^ ^)^ for methodology.

¶ Non-milk extrinsic sugars – added sugars, sugar in fruit juice and half of the sugar naturally present in fruit that is canned, stewed, dried or used in preserves.

** NSP as measured by the Englyst method.

Analysis within equivalised income quintiles (Table 2 and online Supplementary Tables S3 and S4) showed that fruit consumption in the two highest-income quintiles (quintiles 4 and 5) was similar in Scotland and England but significantly lower in Scotland in the two lowest-income quintiles. Total red and processed meat consumption was only significantly higher in Scotland than in England in the two lowest-income quintiles. The processed meat components (i.e. bacon and ham and other red meat products) showed a similar pattern with significantly higher consumption in Scotland in the lowest-income quintiles (although for bacon and ham this was only significantly higher in the lowest-income quintile). Saturated fat as a percentage of food energy was higher in Scotland in all but the highest-income quintile. The consumption of whole milk was only significantly higher in the two lowest-income quintiles, and the consumption of butter was significantly higher in all but the highest-income quintile. For NMES, this was only significantly higher in Scotland in income quintiles 2 and 3 (i.e. the second and third lowest). Fibre intake was lower in Scotland for the lowest 3 income quintiles

**Table 2:** Consumption of 2013 Scottish Dietary Goal foods and nutrients, by equivalised income quintile (Q), 2001 to 2012 combined, Expenditure and Food Survey/Living Costs and Food Survey data (Mean values and 99 % confidence intervals)

	Equivalised income quintile	Scotland†		England†		
Foods or nutrients	(1 lowest, 5 highest)	Mean‡	99 % CI	Mean‡	99 % CI	*P*
Energy (MJ/d)	1	8·4	8·1, 8·7	8·3	8·2, 8·4	0·553
	2	8·5	8·3, 8·8	8·5	8·4, 8·6	0·680
	3	8·4	8·1, 8·6	8·4	8·3, 8·4	0·952
	4	8·5	8·2, 8·7	8·3	8·2, 8·4	0·072
	5	8·4	8·1, 8·6	8·3	8·2, 8·4	0·230
Fruit and vegetables (g/d)§ ∥	1	237	220, 253	272	267, 278	<0·001*
	2	247	231, 263	286	281, 292	<0·001*
	3	253	237, 269	290	285, 295	<0·001*
	4	280	265, 295	303	298, 308	<0·001*
	5	328	311, 344	353	348, 358	<0·001*
Fruit (g/d)§	1	117	107, 127	131	127, 134	0·001*
	2	124	113, 134	142	138, 145	<0·001*
	3	133	124, 143	143	140, 146	0·012
	4	151	141, 161	154	151, 157	0·465
	5	184	173, 195	188	185, 192	0·345
Vegetables (g/d)∥	1	119	110, 129	142	138, 145	<0·001*
	2	123	114, 133	145	142, 148	<0·001*
	3	120	109, 131	147	143, 150	<0·001*
	4	129	121, 137	149	147, 152	<0·001*
	5	144	135, 153	165	162, 167	<0·001*
Oil-rich fish (g/week)	1	27·7	21·9, 33·6	33·3	31·4, 35·2	0·019
	2	32·1	26·6, 37·5	33·6	31·9, 35·3	0·499
	3	27·3	21·6, 33·0	34·1	32·3, 36·0	0·003*
	4	29·8	24·5, 35·2	34·0	32·3, 35·7	0·056
	5	36·7	30·0, 43·3	44·9	42·9, 47·0	0·002*
Total red and processed meat (g/d)¶	1	64·7	60·6, 68·8	58·4	57·0, 59·7	<0·001*
	2	65·5	61·7, 69·4	60·7	59·5, 61·9	0·002*
	3	63·4	60·0, 66·8	60·7	59·6, 61·8	0·054
	4	61·5	58·1, 65·0	60·3	59·2, 61·4	0·385
	5	57·8	54·4, 61·2	58·6	57·6, 59·7	0·560
% Food energy – fat	1	39·3	38·7, 40·0	39·1	38·9, 39·3	0·332
	2	39·1	38·5, 39·7	39·1	38·9, 39·3	0·957
	3	38·9	38·4, 39·4	38·9	38·7, 39·1	0·911
	4	38·3	37·9, 38·8	38·6	38·5, 38·8	0·115
	5	38·6	38·1, 39·0	38·8	38·6, 38·9	0·263
% Food energy – saturated fat	1	15·6	15·3, 15·9	15·0	14·9, 15·1	<0·001*
	2	15·4	15·1, 15·6	15·0	15·0, 15·1	0·003*
	3	15·3	15·0, 15·5	14·9	14·9, 15·0	<0·001*
	4	15·1	14·8, 15·3	14·8	14·7, 14·9	0·002*
	5	15·0	14·8, 15·3	14·8	14·8, 14·9	0·048
% Food energy – NMES**	1	15·3	14·7, 15·9	15·0	14·8, 15·2	0·289
	2	15·7	15·2, 16·2	15·0	14·8, 15·1	0·001*
	3	15·5	15·1, 16·0	15·0	14·9, 15·2	0·008*
	4	15·1	14·7, 15·5	14·8	14·7, 15·0	0·114
	5	14·5	14·0, 14·9	14·1	14·0, 14·2	0·038
Fibre (NSP) (g/d)††	1	11·6	11·1, 12·1	12·2	12·1, 12·4	0·002*
	2	12·2	11·7, 12·7	12·8	12·6, 12·9	0·002*
	3	12·0	11·5, 12·5	12·8	12·6, 12·9	<0·001*
	4	12·8	12·3, 13·3	13·0	12·8, 13·1	0·298
	5	13·3	12·9, 13·8	13·6	13·5, 13·8	0·129

* *P*<0·01 considered significant.

† Sample size – Scotland=6431 households (Q1–1363, Q2–1271; Q3–1338; Q4–1267; Q5–1192); England=59 958 households (Q1–11 560, Q2–11 700; Q3–11 816; Q4–12 172; Q5–12 710).

‡ Means adjusted by survey year, age of household reference person, age when the household reference person left full-time education.

§ Fruit includes fruit and vegetable juice.

∥ Vegetables include baked beans.

¶ Meat portion only – see appendices 2 and 4 of Barton *et al.* ^(^ ^34^ ^)^ for methodology.

** Non-milk extrinsic sugars – i.e. added sugars, sugar in fruit juice and half of the sugar naturally present in fruit that is canned, stewed, dried or used in preserves.

†† NSP as measured by the Englyst method.

In summary, it was found that the foods and nutrients that should be increased in the diet, e.g. fruit, vegetables, oil-rich fish and fibre, were lower in Scotland and that these inequalities in diet were more pronounced in the lower-income group. Similarly, the nutrients and foods (saturated fat, NMES, processed meat, confectionery and soft drinks) that should be reduced were higher in Scotland with some evidence that for processed meat the regional differences were more apparent in the lower-income quintiles. It was also noted that, compared with England, alcohol intake was significantly higher in Scotland; however, there were no significant differences in income quintiles (online Supplementary Table S4).

## Discussion

Analysis of 12 years of food purchase survey data adjusted for waste, as well as confounding factors such as equivalised income, found lower intakes of fruit and vegetables, oil-rich fish, fibre, vitamins A, D, E and folate and higher intakes of red and processed meat, whole milk, butter, savoury snacks, confectionery, soft drinks, saturated fat and NMES and alcohol in Scotland compared with England. On comparison of differences within equivalised income quintiles, it was found that for some parameters there was no difference between Scotland and England when comparisons were made within the more affluent equivalised income categories for fruit, red and processed meat, vitamin C, fibre and alcohol. This suggests that the poorer diet seen in Scotland compared with England may be primarily between those in lower socio-economic groups.

The review conducted by Chambers *et al.* ^(^ ^14^ ^)^ found limited comparisons of diet between Scotland and England in the published literature. The main differences were lower intakes of fruit and vegetables, fibre and water- and fat-soluble vitamins in Scotland, and higher intakes of Ca, salt and processed meat, suggesting a greater emphasis on animal- *v*. plant-based diets. The overall findings showed that historic differences in the diet between Scotland and England were still apparent when more recent data were analysed. For example, the results of the Health and Lifestyle Survey from 1992^(^ ^26^ ^)^ showed lower consumption of salad, green vegetables and fruit within both non-manual and manual groups in Scotland compared with England, supporting findings from the first Health and Lifestyle Survey of the same participants 7 years earlier^(^ ^27^ ^)^. These differences have persisted in recent years as demonstrated in the analysis of the EFS/LCFS. The impact of lower fruit and vegetable consumption is likely to be compounded by lower intakes of oil-rich fish and higher intakes of alcohol, fibre and processed meat, which were more pronounced within lower-income households in Scotland *v*. England. It is also recognised that dietary intake is poorer in populations with higher levels of multiple co-morbidities^(^ ^17^ ^)^. This finding may be related to both cause and effect and brings into focus dietary support for people living with long-term conditions.

Oyebode *et al.* ^(^ ^28^ ^)^ recently highlighted the potential impact of lower fruit and vegetable consumption using Health Survey for England data. Fruit and vegetable consumption was found to be inversely associated with all-cause, cancer and cardiovascular mortality. Those consuming seven or more portions of fruit and vegetables daily were found to have the lowest risk of mortality from any cause, and consumption of vegetables, salad and fresh or dried fruit was robustly associated with decreased mortality. Given that the risk of mortality is known to be higher in more deprived communities, this raises concern about the combined effects of dietary factors (e.g. impact of low folate and high alcohol intakes)^(^ ^29^ ^,^ ^30^ ^)^ and is a reminder of the importance of focusing on whole-diet approaches rather than single-nutrient programmes (which in terms of supplementation may also be associated with negative health outcomes)^(^ ^31^ ^)^. These dietary differences between Scotland and England appear to be apparent from the early years with data from pre-school children from 1950 and 1992 showing poorer diets in Scotland compared with England^(^ ^32^ ^,^ ^33^ ^)^. Thus, the long-term impact of the poorer Scottish Diet across the life course on health outcomes is well founded.

The EFS and its successor, the LCFS, have been used in Scotland to monitor the Scottish Dietary Targets and subsequently the Revised Scottish Dietary Goals backdated to 2001^(^ ^34^ ^–^ ^36^ ^)^. These surveys show that the Scottish diet has been slow to change despite new policy initiatives^(^ ^6^ ^)^. As the purchase surveys are UK-wide and carried out annually, they were also considered to be one of the major sources of data to compare the diet of Scotland and England.

There are limitations to these types of data in estimating food and nutrient intake. As with all surveys that collect information on food intake, the data are self-reported and therefore likely to be affected by bias, although it is possibly less susceptible to under-reporting and non-response bias than weighed intake dietary surveys^(^ ^37^ ^)^. This is because the individual perceptions of what should be reported are less likely if someone is reporting on household purchases rather than individual consumption. The response rates to this purchase survey have dropped over recent years but are still above 50 %, a figure similar to response rates to other surveys collecting dietary data such as the NDNS (53 % in 2012/2013 and 2013/2014)^(^ ^38^ ^)^, and the Low Income and Diet Survey of (55 % in 2003–2005)(17). This is not optimal but the survey data are weighted to reduce the effect on non-response bias. Assumptions are made in the adjustment for waste using national data on waste rather than individual assessment. As the data are collected at household level, it is not possible to adjust for individual factors such as age, sex, education level and smoking. However, the results presented in this study have been adjusted by the age of the household reference person and the age when the household reference person left school. It should be noted that smokers have been reported to have poorer diets than non-smokers^(^ ^39^ ^,^ ^40^ ^)^ and the current and historic higher prevalence of smoking in Scotland^(^ ^11^ ^,^ ^41^ ^,^ ^42^ ^)^ is likely to compound the impact of poor diet (as smoking may increase nutrient demand). Cultural differences are another factor that might have an influence, such as a greater social norm to make healthier choices^(^ ^43^ ^)^ in England compared with Scotland.

The failure of recent policy initiatives to improve the Scottish diet suggests that education alone is unlikely to produce the necessary impact. In the development of the National Food Policy, the Government Food Leadership group noted that the influence of Scotland’s well-crafted health education programmes aimed at consumers are competing with media and commercial messages^(^ ^44^ ^)^. Furthermore, it is likely that advantaged groups are likely to derive greater benefit from health education compared with poorer groups where change is contingent on action by individuals compared with population-based approaches^(^ ^45^ ^)^. Recent proposals from government agencies such as Food Standards Scotland have called for much wider reaching actions to change dietary behaviours including actions to tackle price and promotions, advertising and marketing, reformulation and taxation recognising that the dietary health of the Scottish population will not change by education alone^(^ ^46^ ^)^. Similarly, Adams *et al.* ^(^ ^47^ ^)^ argued recently that in order to reduce dietary inequalities we must put in place more population-level interventions, which reduce the need for individual decisions (such as fortification and fiscal measures) and support efforts to make these politically and publicly acceptable.

### Conclusions

Analysis showed that the foods and nutrients highlighted in the 2013 Scottish Dietary Goals that should be increased in the diet were lower and (with the exception of fat) those that should be reduced were higher in Scotland compared with England, and that in most cases these inequalities in diet were more pronounced in the lower-income groups.

Analysis of 12-year food purchase survey data, with adjustment for waste (as well as confounding factors such as equivalised income), showed lower intakes of fruit and vegetables, oil-rich fish, fibre, vitamin A, D, E and folate and higher intakes of red and processed meat, whole milk, butter, savoury snacks, confectionery, soft drinks, saturated fat, NMES and alcohol in Scotland compared with England. Comparison of differences within equivalised income quintiles suggested that differences in dietary components known to be related to health outcomes, namely fruit and vegetables, red and processed meat, sugar-containing soft drinks, saturated fat and fibre, were more apparent in those with lower incomes.

A poorer diet in Scotland compared with England, particularly among disadvantaged groups, is likely to be one of the reasons for excess mortality. The current evidence on the continued poor diet in Scotland, particularly in disadvantaged groups, should not be ignored. Identifying effective, culturally appropriate approaches to improve diet across the population and notably in the most deprived areas needs further investment. It is unlikely that we will reduce health and diet inequalities without substantial change in policy at the highest level.

## Supplementary material

For supplementary material accompanying this paper visit http://dx.doi.org/10.1017/S0007114518001435. click here to view supplementary material
