# Supplementary material for: Time to address the double inequality of differences in dietary intake between Scotland and England
Source: Br J Nutr. 2018 Jul 28;120(2):220–6. doi: 10.1017/S0007114518001435 (PMC6088540; doi:10.1017/S0007114518001435)
Supplement: Supplementary file 1 [file S0007114518001435sup001.docx]

Supplementary Tables

Table S1: Mean (with 99% confidence intervals) intake of selected nutrients, 2001 to 2012 combined, EFS/LCFS data (units/person/day)

| **Nutrient** | **Scotland** | | **England** | | **P-value**^*^ |
| --- | --- | --- | --- | --- | --- |
|  | **Mean**^†^ | **99%CI** | **Mean**^†^ | **99% CI** |  |
| % Food Energy - Protein | 12.2 | 12.1, 12.3 | 12.3 | 12.3, 12.3 | ***0.007*** |
| % Food Energy - Carbohydrate | 47.0 | 46.8, 47.3 | 46.8 | 46.7, 46.8 | *0.015* |
| Vitamin A (μg) (Retinol Equivalents) | 781 | 756, 805 | 823 | 816, 831 | ***<0.001*** |
| Folate (μg) | 250 | 246, 254 | 262 | 260, 263 | ***<0.001*** |
| Vitamin C (mg) | 65.5 | 64.1, 66.9 | 66.8 | 66.4, 67.3 | *0.020* |
| Vitamin D (μg) | 2.9 | 2.8, 2.9 | 3.0 | 3.0, 3.0 | ***<0.001*** |
| Vitamin E (mg) | 11.0 | 10.7, 11.3 | 11.4 | 11.4, 11.5 | ***<0.001*** |
| Calcium (mg) | 879 | 866, 891 | 862 | 858, 866 | ***0.002*** |
| Iron (mg) | 10.4 | 10.3, 10.6 | 10.6 | 10.5, 10.6 | *0.128* |
| Potassium (mg) | 2.8 | 2.7, 2.8 | 2.8 | 2.8, 2.8 | ***0.002*** |
| Alcohol (g) | 9.9 | 9.4, 10.4 | 9.3 | 9.2, 9.5 | ***0.008*** |

Sample Size – Scotland = 6431; England = 59,958. ^*^P-values <0.01 (in bold) considered significant; ^†^Means adjusted by survey year, equivalised income quintiles, age of household reference person, age household reference person left full-time education;

Table S2: Mean (with 99% confidence intervals) consumption of additional foods and drinks indicative of diet quality, 2001 to 2012 combined, EFS/LCFS data (g/person/day)

| **Food** | **Scotland** | | **England** | | **P-value^*^** |
| --- | --- | --- | --- | --- | --- |
|  | **Mean**^†^ | **99%CI** | **Mean**^†^ | **99%CI** |  |
| Total Bread | 98.4 | 96.6, 100 | 89.3 | 88.8, 89.9 | ***<0.001*** |
| Brown/Wholemeal Bread | 21.1 | 20.2, 22.0 | 20.4 | 20.1, 20.7 | *0.062* |
| Total Breakfast Cereal | 20.4 | 19.5, 21.3 | 20.6 | 20.4, 20.9 | *0.505* |
| High Fibre Breakfast Cereal | 11.4 | 10.7, 12.2 | 12.5 | 12.3, 12.7 | ***<0.001*** |
| Cakes and Pastries | 17.0 | 16.3, 17.7 | 18.4 | 18.1, 18.6 | ***<0.001*** |
| Sweet Biscuits | 22.0 | 21.2, 22.8 | 20.1 | 19.9, 20.4 | ***<0.001*** |
| Cakes, Sweet Biscuits and Pastries | 39.0 | 37.9, 40.2 | 38.5 | 38.1, 38.9 | *0.257* |
| Sugar and Preserves | 18.1 | 17.1, 19.0 | 18.7 | 18.4, 19.0 | *0.085* |
| Chocolate Confectionery | 14.4 | 13.8, 15.0 | 12.5 | 12.3, 12.7 | ***<0.001*** |
| Sugar Confectionery | 7.1 | 6.7, 7.5 | 5.9 | 5.8, 6.0 | ***<0.001*** |
| Total Confectionery | 21.5 | 20.7, 22.3 | 18.5 | 18.2, 18.7 | ***<0.001*** |
| Sugar Containing Soft Drinks | 218 | 210, 225 | 182 | 180, 184 | ***<0.001*** |
| Sugar Free Soft Drinks | 100 | 95.0, 105 | 70.3 | 68.7, 72.0 | ***<0.001*** |
| Total Soft Drinks | 318 | 309, 327 | 252 | 249, 255 | ***<0.001*** |
| Bacon and Ham | 12.1 | 11.6, 12.5 | 11.2 | 11.1, 11.4 | ***<0.001*** |
| Other Red Meat Products^‡§^ | 27.6 | 26.8, 28.4 | 24.2 | 23.9, 24.5 | ***<0.001*** |
| Butter | 6.5 | 6.2, 6.9 | 5.3 | 5.1, 5.4 | ***<0.001*** |
| Whole Milk | 66.5 | 62.7, 70.4 | 59.1 | 57.9, 60.4 | ***<0.001*** |
| Semi-skimmed Milk | 131 | 127, 136 | 124 | 122, 125 | ***<0.001*** |
| Skimmed Milk | 14.2 | 11.8, 16.6 | 21.6 | 20.8, 22.4 | ***<0.001*** |
| Total Milk | 232 | 226, 238 | 227 | 225, 229 | *0.033* |
| White Fish | 87.4 | 83.0, 91.8 | 91.7 | 90.3, 93.1 | *0.017* |
| Fresh Potatoes | 54.0 | 51.4, 56.6 | 60.8 | 59.9, 61.6 | ***<0.001*** |
| Processed Potatoes | 29.5 | 28.5, 30.5 | 26.9 | 26.6, 27.2 | ***<0.001*** |
| Savoury Snacks | 13.0 | 12.6, 13.4 | 11.0 | 10.8, 11.1 | ***<0.001*** |

Sample Size – Scotland = 6431; England = 59,958. ^*^P-values <0.01 (in bold) considered significant; **^†^**Means adjusted by survey year, equivalised income quintiles, age of household reference person, age household reference person left full-time education; ^‡^Meat portion only – see appendices 2 & 4 of Barton and Wrieden, 2012 for methodology; ^§^Other Red Meat products includes the meat portion of sausages, meat pies, corned beef, burgers and pate and is a component of total red meat.

Barton KL, Wrieden WL (2012) Estimation of food and nutrient intakes from food survey data in Scotland 2001-2009. <http://tna.europarchive.org/20141103165934/http://www.foodbase.org.uk//admintools/reportdocuments/749-1-1324_Final_Report_2001-2009.pdf> (accessed 29 June 2017).

Table S3: Mean (with 99% confidence intervals) intake of selected nutrients, by equivalised income quintile, 2001 to 2012 combined, EFS/LCFS data (units/person/day)

| **Nutrient** | **Equivalised Income Quintile**  **(1 lowest, 5 highest)** | **Scotland** | | **England** | | **P-value^*^** |
| --- | --- | --- | --- | --- | --- | --- |
|  |  | **Mean**^†^ | **99% CI** | **Mean**^†^ | **99% CI** |  |
| **% Food Energy - Protein** | 1 | 12.7 | 12.5, 13.0 | 12.6 | 12.5, 12.7 | *0.166* |
|  | 2 | 12.3 | 12.0, 12.6 | 12.4 | 12.3, 12.4 | *0.662* |
|  | 3 | 12.1 | 11.9, 12.3 | 12.3 | 12.2, 12.4 | *0.024* |
|  | 4 | 12.1 | 11.9, 12.3 | 12.2 | 12.1, 12.3 | *0.155* |
|  | 5 | 11.8 | 11.6, 12.0 | 12.1 | 12.0, 12.2 | ***0.003*** |
| **% Food Energy - Carbohydrate** | 1 | 46.6 | 46.0, 47.3 | 46.9 | 46.7, 47.1 | *0.372* |
|  | 2 | 46.9 | 46.3, 47.5 | 46.7 | 46.6, 46.9 | *0.536* |
|  | 3 | 47.0 | 46.5, 47.5 | 46.8 | 46.7, 47.0 | *0.448* |
|  | 4 | 47.4 | 46.9, 47.9 | 46.9 | 46.8, 47.1 | *0.024* |
|  | 5 | 46.9 | 46.4, 47.4 | 46.4 | 46.2, 46.5 | *0.014* |
| **Vitamin A (μg) (Retinol Equivalents)** | 1 | 777 | 713, 842 | 829 | 808, 849 | *0.051* |
|  | 2 | 801 | 739, 864 | 855 | 835, 875 | *0.034* |
|  | 3 | 779 | 723, 834 | 827 | 809, 845 | *0.034* |
|  | 4 | 769 | 722, 817 | 801 | 785, 816 | *0.104* |
|  | 5 | 806 | 758, 853 | 836 | 821, 850 | *0.121* |
| **Folate (μg)** | 1 | 239 | 229, 248 | 251 | 247, 254 | ***0.003*** |
|  | 2 | 249 | 239, 258 | 262 | 259, 265 | ***0.001*** |
|  | 3 | 244 | 235, 253 | 261 | 258, 263 | ***<0.001*** |
|  | 4 | 256 | 248, 265 | 264 | 261, 267 | *0.029* |
|  | 5 | 268 | 259, 278 | 277 | 274, 280 | *0.027* |
| **Vitamin C (mg)** | 1 | 56.5 | 53.4, 59.6 | 59.5 | 58.5, 60.5 | *0.017* |
|  | 2 | 60.5 | 57.3, 63.6 | 64.2 | 63.2, 65.2 | ***0.004*** |
|  | 3 | 63.4 | 60.5, 66.3 | 65.2 | 64.2, 66.1 | *0.145* |
|  | 4 | 69.6 | 66.5, 72.6 | 68.6 | 67.7, 69.6 | *0.452* |
|  | 5 | 78.2 | 74.8, 81.6 | 77.9 | 76.9, 79.0 | *0.847* |
| **Vitamin D (μg)** | 1 | 2.9 | 2.8, 3.1 | 3.1 | 3.0, 3.1 | *0.029* |
|  | 2 | 3.0 | 2.8, 3.2 | 3.1 | 3.1, 3.2 | *0.031* |
|  | 3 | 2.8 | 2.7, 3.0 | 3.0 | 3.0, 3.1 | ***<0.001*** |
|  | 4 | 2.9 | 2.7, 3.0 | 3.0 | 2.9, 3.0 | *0.050* |
|  | 5 | 2.8 | 2.7, 3.0 | 3.0 | 2.9, 3.0 | ***0.006*** |
| **Vitamin E (mg)** | 1 | 10.9 | 10.1, 11.6 | 11.6 | 11.4, 11.9 | *0.011* |
|  | 2 | 11.3 | 10.5, 12.0 | 11.8 | 11.6, 12.1 | *0.056* |
|  | 3 | 11.1 | 10.5, 11.7 | 11.5 | 11.3, 11.7 | *0.098* |
|  | 4 | 10.8 | 10.3, 11.4 | 11.3 | 11.1, 11.4 | *0.055* |
|  | 5 | 10.9 | 10.5, 11.4 | 11.1 | 11.0, 11.3 | *0.307* |
| **Calcium (mg)** | 1 | 895 | 863, 927 | 860 | 849, 870 | ***0.007*** |
|  | 2 | 894 | 863, 925 | 885 | 875, 894 | *0.454* |
|  | 3 | 873 | 846, 901 | 864 | 855, 873 | *0.411* |
|  | 4 | 882 | 855, 908 | 861 | 852, 869 | *0.052* |
|  | 5 | 870 | 843, 897 | 860 | 852, 868 | *0.359* |
| **Iron (mg)** | 1 | 10.0 | 9.6, 10.3 | 10.0 | 9.9, 10.2 | *0.647* |
|  | 2 | 10.3 | 9.9, 10.7 | 10.5 | 10.3, 10.6 | *0.280* |
|  | 3 | 10.3 | 9.9, 10.6 | 10.5 | 10.4, 10.6 | *0.089* |
|  | 4 | 10.8 | 10.4, 11.1 | 10.7 | 10.6, 10.9 | *0.816* |
|  | 5 | 11.1 | 10.8, 11.5 | 11.2 | 11.1, 11.3 | *0.683* |
| **Potassium (mg)** | 1 | 2.7 | 2.6, 2.8 | 2.7 | 2.7, 2.7 | *0.341* |
|  | 2 | 2.8 | 2.7, 2.9 | 2.8 | 2.8, 2.9 | *0.053* |
|  | 3 | 2.7 | 2.7, 2.8 | 2.8 | 2.8, 2.9 | *0.026* |
|  | 4 | 2.8 | 2.7, 2.9 | 2.8 | 2.8, 2.9 | *0.366* |
|  | 5 | 2.9 | 2.8, 3.0 | 2.9 | 2.9, 3.0 | *0.366* |
| **Alcohol (g)** | 1 | 7.6 | 6.6, 8.6 | 6.6 | 6.3, 7.0 | *0.018* |
|  | 2 | 8.8 | 7.8, 9.8 | 7.8 | 7.4, 8.1 | *0.012* |
|  | 3 | 9.9 | 8.9, 10.9 | 9.3 | 8.9, 9.6 | *0.111* |
|  | 4 | 10.9 | 9.8, 12.0 | 10.9 | 10.6, 11.3 | *0.983* |
|  | 5 | 12.6 | 11.4, 13.8 | 12.4 | 12.0, 12.8 | *0.653* |

Sample Size –Scotland = 6431 households (Q1 – 1363, Q2 – 1271; Q3 – 1338; Q4 – 1267; Q5 – 1192); England = 59,958 households (Q1 – 11,560, Q2 – 11,700; Q3 – 11,816; Q4 – 12,172; Q5 – 12,710). ^*^P-values <0.01 (in bold) considered significant; ^†^Means adjusted by survey year, age of household reference person, age household reference person left full-time education.

Table S4: Mean (with 99% confidence intervals) consumption of additional foods and drinks indicative of diet quality, by equivalised income quintile, 2001 to 2012 combined, EFS/LCFS data (g/person/day with the exception of fish g/person/week)

| **Food** | **Equivalised Income Quintile (1 lowest, 5 highest)** | **Scotland** | | **England** | | **P-value^*^** |
| --- | --- | --- | --- | --- | --- | --- |
|  |  | **Mean**^†^ | **99% CI** | **Mean**^†^ | **99% CI** |  |
| Total Bread | 1 | 102 | 97.9, 107 | 90.0 | 88.5, 91.4 | ***<0.001*** |
|  | 2 | 102 | 97.4, 106 | 89.6 | 88.2, 90.9 | ***<0.001*** |
|  | 3 | 97.9 | 94.1, 102 | 90.0 | 88.8, 91.3 | ***<0.001*** |
|  | 4 | 99.0 | 95.5, 103 | 90.5 | 89.3, 91.6 | ***<0.001*** |
|  | 5 | 93.5 | 90.0, 97.0 | 87.8 | 86.7, 88.9 | ***<0.001*** |
| Brown/Wholemeal Bread | 1 | 20.1 | 17.8, 22.4 | 19.8 | 19.1, 20.6 | *0.768* |
|  | 2 | 22.5 | 20.3, 24.6 | 19.9 | 19.2, 20.5 | ***0.003*** |
|  | 3 | 20.7 | 18.8, 22.6 | 19.7 | 19.0, 20.3 | *0.189* |
|  | 4 | 21.2 | 19.3, 23.2 | 20.9 | 20.3, 21.5 | *0.643* |
|  | 5 | 22.3 | 20.4, 24.2 | 22.8 | 22.2, 23.4 | *0.509* |
| Total Breakfast Cereal | 1 | 19.3 | 17.3, 21.3 | 18.9 | 18.2, 19.5 | *0.589* |
|  | 2 | 19.8 | 17.8, 21.8 | 19.9 | 19.2, 20.5 | *0.947* |
|  | 3 | 19.3 | 17.5, 21.2 | 20.2 | 19.6, 20.8 | *0.243* |
|  | 4 | 21.8 | 19.9, 23.8 | 21.7 | 21.1, 22.3 | *0.865* |
|  | 5 | 22.6 | 20.6, 24.7 | 23.4 | 22.8, 24.0 | *0.369* |
| High Fibre Breakfast Cereal | 1 | 11.1 | 9.4, 12.7 | 11.4 | 10.9, 12.0 | *0.594* |
|  | 2 | 10.9 | 9.3, 12.6 | 12.0 | 11.5, 12.5 | *0.101* |
|  | 3 | 10.5 | 9.0, 12.0 | 11.9 | 11.4, 12.3 | *0.022* |
|  | 4 | 12.3 | 10.7, 13.9 | 13.0 | 12.5, 13.5 | *0.293* |
|  | 5 | 13.3 | 11.6, 15.1 | 15.0 | 14.5, 15.6 | *0.015* |
| Cakes and Pastries | 1 | 16.6 | 14.9, 18.3 | 18.4 | 17.8, 18.9 | *0.011* |
|  | 2 | 17.7 | 16.0, 19.5 | 19.9 | 19.3, 20.4 | ***0.003*** |
|  | 3 | 16.3 | 14.8, 17.9 | 18.7 | 18.2, 19.2 | ***<0.001*** |
|  | 4 | 17.9 | 16.4, 19.4 | 18.3 | 17.8, 18.7 | *0.503* |
|  | 5 | 17.4 | 15.9, 18.9 | 17.8 | 17.3, 18.2 | *0.529* |
| Sweet Biscuits | 1 | 23.8 | 21.9, 25.7 | 20.7 | 20.0, 21.3 | ***<0.001*** |
|  | 2 | 23.7 | 21.8, 25.6 | 21.9 | 21.3, 22.5 | *0.019* |
|  | 3 | 22.0 | 20.3, 23.8 | 21.2 | 20.6, 21.7 | *0.223* |
|  | 4 | 21.5 | 19.9, 23.1 | 20.0 | 19.5, 20.5 | *0.021* |
|  | 5 | 19.8 | 18.4, 21.3 | 17.5 | 17.1, 18.0 | ***<0.001*** |
| Cakes, Sweet Biscuits and Pastries | 1 | 40.4 | 37.5, 43.2 | 39.0 | 38.1, 39.9 | *0.238* |
|  | 2 | 41.5 | 38.6, 44.3 | 41.8 | 40.9, 42.7 | *0.778* |
|  | 3 | 38.3 | 35.8, 40.9 | 39.8 | 39.0, 40.6 | *0.161* |
|  | 4 | 39.4 | 37.0, 41.8 | 38.3 | 37.5, 39.1 | *0.272* |
|  | 5 | 37.2 | 34.9, 39.5 | 35.3 | 34.6, 36.0 | *0.036* |
| Sugar and Preserves | 1 | 24.6 | 21.8, 27.3 | 24.7 | 23.8, 25.6 | *0.888* |
|  | 2 | 22.1 | 19.6, 24.5 | 22.3 | 21.5, 23.0 | *0.855* |
|  | 3 | 17.1 | 15.0, 19.2 | 18.6 | 18.0, 19.3 | *0.069* |
|  | 4 | 14.6 | 12.8, 16.4 | 15.6 | 15.1, 16.2 | *0.151* |
|  | 5 | 13.2 | 11.5, 14.9 | 13.5 | 13.0, 14.0 | *0.680* |
| Chocolate Confectionery | 1 | 11.7 | 10.3, 13.2 | 11.2 | 10.7, 11.7 | *0.360* |
|  | 2 | 13.8 | 12.3, 15.2 | 12.2 | 11.8, 12.7 | *0.011* |
|  | 3 | 15.6 | 14.2, 17.0 | 13.2 | 12.8, 13.7 | ***<0.001*** |
|  | 4 | 15.4 | 14.1, 16.7 | 13.1 | 12.7, 13.6 | ***<0.001*** |
|  | 5 | 15.2 | 13.8, 16.7 | 13.1 | 12.7, 13.6 | ***<0.001*** |
| Sugar Confectionery | 1 | 6.9 | 5.9, 7.8 | 6.3 | 6.0, 6.6 | *0.105* |
|  | 2 | 7.7 | 6.8, 8.6 | 6.5 | 6.3, 6.8 | ***0.002*** |
|  | 3 | 7.3 | 6.5, 8.1 | 6.0 | 5.8, 6.3 | ***<0.001*** |
|  | 4 | 6.9 | 6.1, 7.7 | 5.7 | 5.5, 6.0 | ***<0.001*** |
|  | 5 | 6.7 | 6.0, 7.5 | 5.2 | 5.0, 5.4 | ***<0.001*** |
| Total Confectionery | 1 | 18.6 | 16.7, 20.5 | 17.4 | 16.8, 18.0 | *0.124* |
|  | 2 | 21.5 | 19.6, 23.3 | 18.8 | 18.2, 19.4 | ***<0.001*** |
|  | 3 | 22.9 | 21.2, 24.6 | 19.2 | 18.7, 19.8 | ***<0.001*** |
|  | 4 | 22.3 | 20.7, 24.0 | 18.9 | 18.3, 19.4 | ***<0.001*** |
|  | 5 | 22.0 | 20.3, 23.7 | 18.3 | 17.8, 18.9 | ***<0.001*** |
| Sugar Containing Soft Drinks | 1 | 195 | 178, 212 | 164 | 158, 170 | ***<0.001*** |
|  | 2 | 220 | 202, 238 | 178 | 173, 184 | ***<0.001*** |
|  | 3 | 243 | 226, 259 | 194 | 188, 199 | ***<0.001*** |
|  | 4 | 226 | 210, 242 | 195 | 190, 200 | ***<0.001*** |
|  | 5 | 200 | 184, 215 | 172 | 167, 177 | ***<0.001*** |
| Sugar Free Soft Drinks | 1 | 73.4 | 62.5, 84.3 | 52.7 | 49.1, 56.2 | ***<0.001*** |
|  | 2 | 86.8 | 75.8, 97.8 | 61.0 | 57.6, 64.5 | ***<0.001*** |
|  | 3 | 102 | 90.6, 113 | 73.0 | 69.3, 76.6 | ***<0.001*** |
|  | 4 | 118 | 106, 130 | 82.0 | 78.2, 85.7 | ***<0.001*** |
|  | 5 | 115 | 103, 127 | 82.0 | 78.4, 85.6 | ***<0.001*** |
| Total Soft Drinks | 1 | 268 | 248, 289 | 217 | 210, 223 | ***<0.001*** |
|  | 2 | 307 | 286, 328 | 239 | 233, 246 | ***<0.001*** |
|  | 3 | 344 | 324, 365 | 267 | 260, 273 | ***<0.001*** |
|  | 4 | 344 | 324, 364 | 277 | 271, 283 | ***<0.001*** |
|  | 5 | 315 | 295, 335 | 254 | 248, 260 | ***<0.001*** |
| Bacon and Ham | 1 | 12.0 | 10.9, 13.1 | 10.6 | 10.3, 11.0 | ***0.002*** |
|  | 2 | 12.6 | 11.5, 13.7 | 11.6 | 11.2, 11.9 | *0.021* |
|  | 3 | 12.2 | 11.2, 13.2 | 11.6 | 11.3, 11.9 | *0.128* |
|  | 4 | 12.5 | 11.5, 13.5 | 11.6 | 11.3, 11.9 | *0.034* |
|  | 5 | 11.8 | 10.8, 12.7 | 11.3 | 11.0, 11.6 | *0.225* |
| Other Red Meat Products^‡§^ | 1 | 29.9 | 27.8, 32.0 | 24.2 | 23.5, 24.9 | ***<0.001*** |
|  | 2 | 29.6 | 27.7, 31.4 | 24.4 | 23.8, 24.9 | ***<0.001*** |
|  | 3 | 28.3 | 26.6, 30.0 | 25.1 | 24.5, 25.6 | ***<0.001*** |
|  | 4 | 27.6 | 25.9, 29.3 | 24.6 | 24.1, 25.2 | ***<0.001*** |
|  | 5 | 23.6 | 22.0, 25.2 | 22.8 | 22.3, 23.3 | *0.197* |
| Butter | 1 | 7.3 | 6.4, 8.3 | 5.8 | 5.5, 6.1 | ***<0.001*** |
|  | 2 | 7.5 | 6.6, 8.5 | 5.6 | 5.3, 5.9 | ***<0.001*** |
|  | 3 | 6.2 | 5.4, 7.0 | 5.1 | 4.8, 5.3 | ***0.001*** |
|  | 4 | 6.3 | 5.5, 7.0 | 4.7 | 4.5, 5.0 | ***<0.001*** |
|  | 5 | 6.1 | 5.3, 6.8 | 5.5 | 5.3, 5.7 | *0.063* |
| Whole Milk | 1 | 104 | 92.9, 115 | 82.2 | 78.6, 85.9 | ***<0.001*** |
|  | 2 | 82.7 | 72.5, 92.9 | 69.8 | 66.6, 73.0 | ***0.002*** |
|  | 3 | 64.0 | 55.9, 72.1 | 58.0 | 55.3, 60.6 | *0.066* |
|  | 4 | 47.8 | 40.5, 55.1 | 46.6 | 44.3, 49.0 | *0.687* |
|  | 5 | 37.6 | 30.9, 44.3 | 37.5 | 35.4, 39.5 | *0.959* |
| Semi-skimmed Milk | 1 | 130 | 118, 142 | 126 | 122, 130 | *0.477* |
|  | 2 | 138 | 125, 150 | 136 | 132, 140 | *0.737* |
|  | 3 | 134 | 123, 145 | 124 | 120, 128 | *0.030* |
|  | 4 | 134 | 124, 143 | 121 | 118, 125 | ***0.002*** |
|  | 5 | 126 | 116, 135 | 117 | 114, 120 | *0.019* |
| Skimmed Milk | 1 | 15.4 | 9.7, 21.2 | 20.0 | 18.1, 21.9 | *0.050* |
|  | 2 | 14.4 | 8.1, 20.7 | 22.9 | 20.9, 24.9 | ***0.001*** |
|  | 3 | 11.1 | 5.6, 16.5 | 22.2 | 20.4, 23.9 | ***<0.001*** |
|  | 4 | 14.4 | 9.5, 19.2 | 21.7 | 20.1, 23.2 | ***<0.001*** |
|  | 5 | 17.9 | 12.9, 22.9 | 22.9 | 21.3, 24.4 | *0.014* |
| Total Milk | 1 | 267 | 252, 283 | 257 | 252, 262 | *0.088* |
|  | 2 | 256 | 240, 272 | 256 | 251, 261 | *0.958* |
|  | 3 | 232 | 219, 244 | 226 | 221, 230 | *0.250* |
|  | 4 | 214 | 203, 226 | 209 | 205, 212 | *0.211* |
|  | 5 | 198 | 186, 210 | 195 | 191, 199 | *0.525* |
| White Fish | 1 | 79.6 | 68.7, 90.4 | 88.8 | 85.2, 92.3 | *0.038* |
|  | 2 | 88.2 | 78.3, 98.2 | 89.9 | 86.8, 93.1 | *0.673* |
|  | 3 | 83.8 | 74.1, 93.5 | 88.2 | 85.1, 91.4 | *0.262* |
|  | 4 | 89.0 | 80.1, 97.8 | 91.8 | 89.0, 94.6 | *0.434* |
|  | 5 | 99.7 | 89.8, 110 | 104 | 101, 107 | *0.285* |
| Fresh Potatoes | 1 | 61.7 | 54.6, 68.7 | 65.5 | 63.2, 67.8 | *0.185* |
|  | 2 | 61.8 | 55.3, 68.3 | 67.5 | 65.5, 69.6 | *0.030* |
|  | 3 | 52.4 | 46.6, 58.2 | 62.6 | 60.7, 64.5 | ***<0.001*** |
|  | 4 | 51.2 | 46.0, 56.4 | 58.3 | 56.6, 59.9 | ***0.001*** |
|  | 5 | 46.3 | 41.7, 51.0 | 52.8 | 51.3, 54.2 | ***0.001*** |
| Processed Potatoes | 1 | 27.7 | 25.2, 30.2 | 25.6 | 24.8, 26.4 | *0.033* |
|  | 2 | 30.1 | 27.7, 32.4 | 27.1 | 26.4, 27.8 | ***0.002*** |
|  | 3 | 32.0 | 29.8, 34.2 | 29.3 | 28.6, 30.0 | ***0.003*** |
|  | 4 | 31.2 | 29.1, 33.3 | 28.5 | 27.9, 29.2 | ***0.001*** |
|  | 5 | 25.8 | 24.0, 27.7 | 23.6 | 23.0, 24.1 | ***0.002*** |
| Savoury Snacks | 1 | 10.5 | 9.5, 11.6 | 8.9 | 8.6, 9.2 | ***<0.001*** |
|  | 2 | 11.2 | 10.2, 12.1 | 10.4 | 10.1, 10.7 | *0.049* |
|  | 3 | 14.0 | 13.1, 14.9 | 11.7 | 11.4, 12.0 | ***<0.001*** |
|  | 4 | 14.6 | 13.7, 15.5 | 12.2 | 11.9, 12.5 | ***<0.001*** |
|  | 5 | 14.0 | 13.2, 14.9 | 11.3 | 11.1, 11.6 | ***<0.001*** |

Sample Size –Scotland = 6431 households (Q1 – 1363, Q2 – 1271; Q3 – 1338; Q4 – 1267; Q5 – 1192); England = 59,958 households (Q1 – 11,560, Q2 – 11,700; Q3 – 11,816; Q4 – 12,172; Q5 – 12,710). ^*^P-values <0.01 (in bold) considered significant; ^†^Means adjusted by survey year, age of household reference person, age household reference person left full-time education; ^‡^Meat portion only – see appendices 2 & 4 of Barton and Wrieden, 2012 for methodology; ^§^Other Red Meat products includes the meat portion of sausages, meat pies, corned beef, burgers and pate and is a component of total red meat.

Barton KL, Wrieden WL (2012) Estimation of food and nutrient intakes from food survey data in Scotland 2001-2009. <http://tna.europarchive.org/20141103165934/http://www.foodbase.org.uk//admintools/reportdocuments/749-1-1324_Final_Report_2001-2009.pdf> (accessed 29 June 2017).
